# Clinical experience of the attending doctor matters: diagnosing anastomotic leakage after colorectal surgery

**DOI:** 10.1007/s00464-026-12729-1

**Published:** 2026-04-08

**Authors:** M. Cats, L. G. Magermans, A. A. W. van Geloven, H. C. van Santvoort, E. C. E. Wassenaar, J. D. J. Plate, D. Boerma

**Affiliations:** 1 Department of Surgery, St. Antonius Hospital Nieuwegein, Nieuwegein, The Netherlands; 2 Department of Surgery, Tergooi Medical Center Hilversum, Hilversum, The Netherlands; 3 Department of Surgery, University Medical Center Utrecht (UMC Utrecht), Antonius Hospital Nieuwegein, Nieuwegein, The Netherlands; 4 Department of Surgery, Canisius Wilhemina Ziekenhuis Nijmegen, Nijmegen, The Netherlands

**Keywords:** Anastomotic leakage, Colorectal surgery, Clinical experience, Diagnostic accuracy

## Abstract

**Background:**

Clinical assessment is vital in surgical decision-making, and clinical experience is thought to enhance diagnostic judgment. However, the impact of clinical experience on postoperative assessments in surgical care remains unclear. This is particularly critical for timely diagnosing anastomotic leakage after colorectal surgery, where delayed recognition can have severe consequences. This study evaluates to what extent the level of clinician experience influences the accuracy of clinical assessments in diagnosing anastomotic leakage.

**Methods:**

In this prospective multicenter study, clinicians daily estimated the probability (0–100) of anastomotic leakage following colorectal surgery for every patient between 2013 and 2019 in two large teaching hospitals in The Netherlands. Assessors were grouped by experience level as junior doctors and colorectal surgeons. Diagnostic accuracy per group was evaluated via Receiver Operating Characteristics (ROC) analysis, and calibration was assessed using a calibration plot. Missing data were imputed through multiple imputation.

**Results:**

127 (9.5%) of 1336 patients developed anastomotic leakage. The area under the curve for the clinical assessment for anastomotic leakage by junior doctors was 0.87 (95% CI 0.82–0.92) as compared to 0.95 (95% CI 0.91–0.99) for colorectal surgeons (*p* = 0.02). Surgeons appeared to demonstrate superior calibration compared to junior doctors.

**Conclusion:**

Clinical experience enhances the accuracy of clinical assessments in diagnosing anastomotic leakage after colorectal surgery. These findings underscore the importance of involving senior clinicians in postoperative care and highlight the need for targeted training of junior doctors.

**Graphical Abstract:**

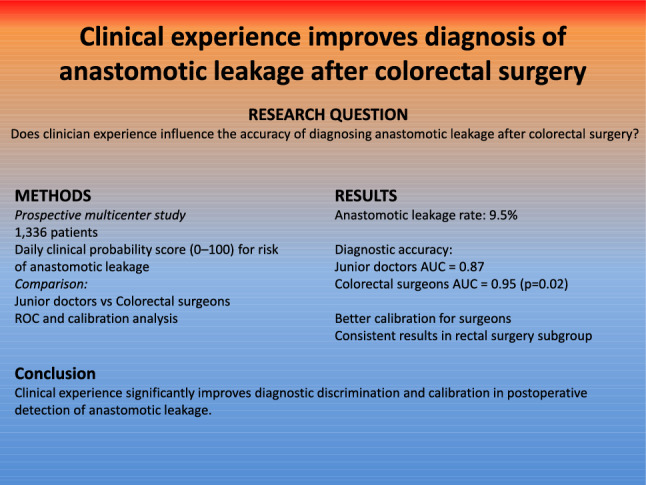

Clinical assessments remain central to medical and surgical decision-making. Despite advances in diagnostic algorithms and the exponential rise in imaging, a clinician’s judgment continues to guide care and relies on a physician’s experience [1, 2]. However, the role of clinical experience in the clinical assessment remains debated. While some evidence suggests that seniority does not necessarily improve diagnostic accuracy of physical examination in acute care settings, other studies have shown the opposite [3, 4]. For instance, physicians with ten or fewer years of experience have been shown to request significantly more inappropriate MRI scans for shoulder, knee, and back pain compared to their more experienced colleagues [3, 4]. At the same time, concerns have been raised that traditional clinical skills are deteriorating in modern medicine, an alarming trend that may compromise patient care [5]. Whether these findings extend to surgical care remains unknown. Yet, the impact of clinical experience may be particularly relevant in high-risk postoperative settings, where subtle clinical changes can signal life-threatening complications. Anastomotic leakage after colorectal surgery provides a valuable model to explore the role of clinical experience, as it is a potentially life-threatening complication whose timely recognition depends heavily on the clinician’s bedside assessment [6–10]. Early diagnosis is essential, yet challenging, as clinical signs are often subtle or nonspecific. While structured tools such as the Dutch Leakage (DULK) score aim to standardize evaluation, bedside assessments remain subjective and are likely influenced by a clinician’s level of experience [11, 12]. Surprisingly, it remains unclear whether the clinical suspicion of a junior doctor carries the same diagnostic value as that of a senior physician in postoperative care. Clarifying this relationship may enable more targeted supervision, reduce unnecessary abdominal CT scans, and support the integration of experience-weighted assessments into structured postoperative care pathways.

Therefore, this study aimed to assess whether clinical experience influences the diagnostic accuracy of the clinical assessment for anastomotic leakage, by comparing junior doctors and colorectal surgeons.

## Methods

### Study design

This prospective, multicenter observational study used a prespecified and published study protocol [13]. It was conducted at the surgical department of the St Antonius Hospital in Nieuwegein and Tergooi Hospital in Hilversum, two large non-academic teaching hospitals in The Netherlands. This study was approved by the medical ethical committee of the St Antonius Hospital, which determined that informed consent was not required. We adhered to the Strengthening the Reporting of Observational Studies in Epidemiology (STROBE) guidelines [14].

### Participants

Patients aged ≥ 18 years who underwent elective colorectal surgery with a primary intestinal anastomosis (i.e., ileo-colic, colonic, or colorectal) between October 2013 and May 2019 were eligible for inclusion. Patients undergoing emergency surgery for colonic perforation were excluded. The study population comprises both conventional colorectal surgery and patients who underwent cytoreductive surgery with hyperthermic intraperitoneal chemotherapy (CRS-HIPEC).

### Clinical assessment

For each included patient, the attending physician assigned a subjective probability score (ranging from 0 to 100) estimating the likelihood of anastomotic leakage, solely based on clinical assessment, on a daily basis. This clinical assessment was derived from parameters such as the patient’s overall condition, vital signs, abdominal symptoms, and postoperative course. As part of routine postoperative care, physicians, including junior doctors, were required to assess mental status, the presence of peritoneal signs (yes/no), and bowel function (e.g., passage of stool), which informed the overall clinical judgment. Each patient was assessed once daily by the physician responsible for postoperative care on that day; assessments were not duplicated across clinician groups. Laboratory values, including C-reactive protein and leukocyte count, were deliberately excluded from the clinical assessment to isolate the diagnostic performance of bedside clinical judgment. This approach reflects clinical situations in which laboratory results are unavailable, delayed, or nonspecific, and enhances generalizability across postoperative settings. No formal scoring guidelines were provided, as the aim was to evaluate diagnostic accuracy under routine clinical conditions.

Clinical assessments were defined as the first postoperative evaluation of the day. Surgeons were not exclusively consulted in response to clinical deterioration; rather, assessments reflected routine postoperative care. During weekdays, junior doctors typically performed the first daily assessment, whereas during weekends this role was more often fulfilled by colorectal surgeons.

### Reference standard

Patients were followed for the occurrence of anastomotic leakage during the hospital admission within 30 days after surgery. Anastomotic leakage was defined as the occurrence of anastomotic dehiscence observed during re-laparoscopy or re-laparotomy, or the drainage of pus from a collection in contact with the anastomosis, either through percutaneous or trans anal means, which served as the reference standard for diagnosis. Anastomotic leakage severity was defined according to the International Study Group of Rectal Cancer (ISREC) definition. [15] Due to the intervention-based reference standard, the present study primarily captures clinically relevant anastomotic leakage requiring active therapeutic intervention (ISREC grade B and C). Asymptomatic leaks managed without active intervention (ISREC grade A) could not be reliably identified within the study design. Patients were assessed daily. Patients who developed an anastomotic leakage were considered a positive case on the specific day when it was detected. Patients diagnosed with anastomotic leakage were excluded from further analysis on the days after anastomotic leakage diagnosis. Patients were considered negative on the days before diagnosis.

### Groups stratified by experience

Clinicians were divided into two groups based on their level of experience. The first group consisted of junior doctors, which are doctors not enrolled in a surgical training program, typically in their first or second year after completing a six-year medical curriculum. The second group included colorectal surgeons. Residents in surgical training were excluded from analysis due to the considerable variability in clinical experience within this group (ranging from 1 to 6 years).

Due to the extended inclusion period of five years, a large and dynamic group of clinicians contributed to postoperative assessments. This included several dozen junior doctors rotating through the surgical wards over time, as well as a stable group of board-certified gastrointestinal surgeons. Because clinicians entered and left clinical service during the study period, the exact number of individual assessors per group could not be reliably reconstructed. This reflects routine clinical practice and enhances the generalizability of the findings.

### Statistical analysis

Descriptive statistics were used to summarize baseline characteristics and clinical scores. Normality of continuous variables was assessed visually. Normally distributed variables were reported as means with standard deviations; non-normally distributed variables as medians with interquartile ranges (IQR). Categorical variables were summarized as counts with percentages. Continuous variables were assessed for normality and compared using the *t*-test when normally distributed or the Wilcoxon rank-sum test when non-normally distributed. Categorical variables were compared using the chi-squared test or Fisher’s exact test, as appropriate. Statistical analyses were performed by the authors, with statistical expertise available within the research team.

### Discrimination

Receiver operating characteristic (ROC) curves were constructed for both groups. The area under the curve (AUC) was calculated with 95% CI’s to quantify diagnostic discrimination. Diagnostic accuracy between groups was compared using DeLong’s test.

### Calibration

Calibration, reflecting the agreement between predicted and observed probabilities of anastomotic leakage, was assessed visually using calibration plots for each clinician group. The subjective clinical scores (ranging from 0 to 100) were grouped into five bins (0–20, 20–40, 40–60, 60–80, and 80–100) to enhance interpretability and ensure sufficient observations within each risk category. The observed proportion of anastomotic leakage within each bin was then plotted against the corresponding mean clinical score.

### Sensitivity analysis

Patients were not assessed by both junior doctors and surgeons, which could potentially introduce bias. To evaluate this, the vital signs and clinical scores at the time of assessment were compared between observations made by junior doctors and those made by surgeons, including heart rate, systolic blood pressure, respiratory rate, and temperature.

### Missing data

Missing data occurred when the clinical scores were not completed by the responsible physician. These missing values were assumed to be missing completely at random or missing at random. Missing values in the clinical score were addressed using multiple imputation by chained equations (MICE) with predictive mean matching. The imputation model included patient-level predictors (e.g., age, heart rate, blood pressure, temperature, and clinical symptoms), scores from adjacent days, and the final anastomotic leakage outcome. Twenty imputed datasets were generated, and diagnostic metrics were pooled using Rubin’s rules. To preserve the integrity of group-level comparisons, observations with missing assessor group data were excluded from the analysis.

All statistical analyses were performed using R version 4.3.1 with the ‘mice’ package. A two-sided *p*-value of < 0.05 was considered statistically significant [16, 17].

## Results

Of the 1468 patients initially included, 1336 patients were available for analysis after excluding 8 patients (0.5%) due to postoperative deaths (unrelated to anastomotic leakage) and 124 patients (8.4%) with missing postoperative data. These 1336 patients accounted for 8050 admission days. The median age was 67 years [IQR 59–74], and 717 patients (53.7%) were male. In total, 127 patients (9.5%) developed clinically relevant anastomotic leakage, of which 9 (7.1%) were classified as ISREC grade B and 118 (92.9%) as ISREC grade C. Leakage rates differed according to the distance of the anastomosis from the anal verge: 19.6% for anastomoses < 5 cm, 9.3% for anastomoses between 5 and 10 cm, and 9.0% for anastomoses > 10 cm from the anal verge. Patient characteristics are presented in Table 1.

**Table 1:** Patient characteristics

	*n* = 1336
General characteristics	
Age (median [IQR])	67 [59, 74]
Sex (%)	
Female	619 (46.3)
Male	717 (53.7)
BMI (median [IQR])	26.06 [23.79, 29.04]
Alcohol units per day (median [IQR])	0 [0, 7]
Smoking (%)	
No	1159 (86.8)
Yes	177 (13.2)
Medical history	
ASA score (%)	
ASA 1/2	1113 (83.3)
ASA 3/4	223 (16.7)
Surgical characteristics	
Type of surgery	
Right hemicolectomy	444 (33.2)
Left hemicolectomy	86 (6.4)
Sigmoid resection	297 (22.2)
Low anterior resection	272 (20.4)
Restoration of continuity*	107 (8.0)
Other**	130 (9.7)
CRS-HIPEC (%)	
Yes	64 (4.8)
No	1272 (95.2)
Number of anastomoses (%)	
1	1293 (96.8)
2	41 (3.1)
3	2 (0.1)
Operation time in minutes (median [IQR])	123 [100, 167]
Intraoperative blood loss (median [IQR])	50 [10, 100]
Anastomotic leakage (%)	
Yes	127 (9.5)
No	1209 (90.5)

*ASA* American Society of Anesthesiologists, *BMI* body mass index, *CRS-HIPEC* cytoreductive surgery with hyperthermic intraperitoneal chemotherapy, *IQR* interquartile range

^*^Restoration of continuity refers to reversal of a temporary stoma (e.g., ileostomy or Hartmann reversal) with construction of a colorectal or coloanal anastomosis

^**^Other procedures included procedures not classified as standard colon or rectal resections, such as limited segmental resections, (sub)total colectomies, or procedures involving multiple or atypical anastomoses

The clinical score was assigned 8050 times among the included patients. Junior doctors assigned 6110 scores (75.9%) while surgeons assigned 676 scores (8.4%). Residents in surgical training assigned 993 scores (12.3%) and 271 (3.4%) observations lacked information on the assessor group; these were excluded from analysis. Missing data occurred in 1604 (19.9%) of the scores and were handled using multiple imputation.

### Diagnostic performance

**Fig. 1:**
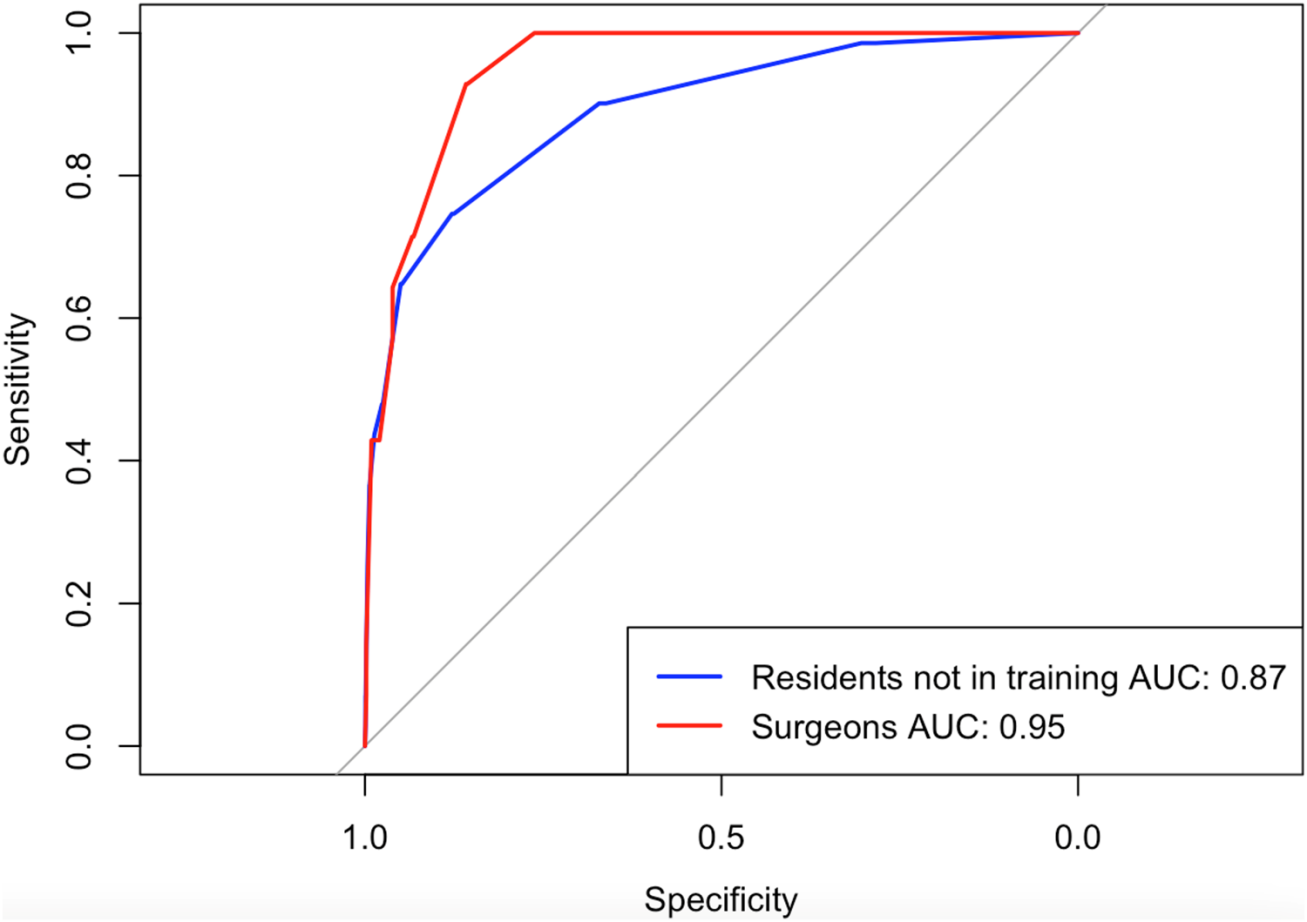
Receiver operating characteristic (ROC) curves for anastomotic leakage. The ROC curves illustrate the diagnostic accuracy of clinical assessment for predicting anastomotic leakage after colorectal surgery. The area under the curve (AUC) is provided for each group. All points on the curve represent sensitivity and 1-specificity for different risk thresholds

The discriminatory performance of the clinical assessment is shown in the ROC curves (Fig. 1). The AUC was 0.87 (95% CI 0.82–0.92) for junior doctors and 0.95 (95% CI 0.91–0.99) for colorectal surgeons (*p* = 0.02). This indicates that colorectal surgeons demonstrated significantly better diagnostic discrimination than junior doctors. A similar difference in diagnostic discrimination was observed in a subgroup analysis restricted to rectal resections (AUC 0.88 vs. 0.96 for junior doctors and colorectal surgeons, respectively).

**Fig. 2:**
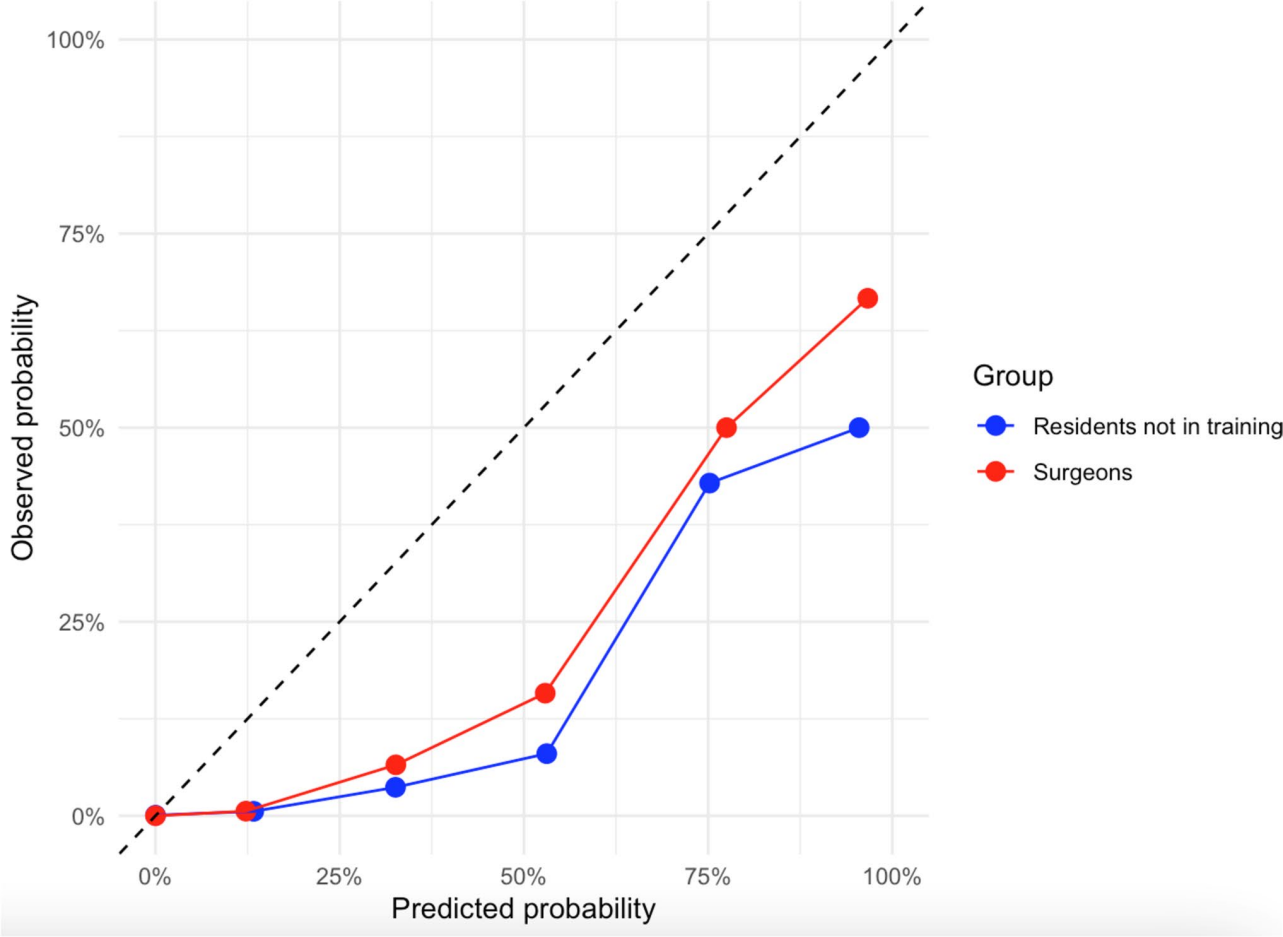
Calibration plots compare predicted probabilities of anastomotic leakage with observed outcomes for assessments performed by junior doctors and colorectal surgeons. Predicted risks were grouped into five categories (0–20, 20–40, 40–60, 60–80, and 80–100). The diagonal line represents perfect agreement between predicted and observed risk. Deviations from this line indicate over- or underestimation of anastomotic leakage risk

Calibration plots (Fig. 2) demonstrated systematic overestimation of the risk of anastomotic leakage across both clinician groups, with surgeons displaying the most accurate predicted probabilities. This was reflected by negative logit calibration intercepts (logit scale) in both groups (− 2.85 for junior doctors and − 2.14 for surgeons), calibration slopes (0.51 for junior doctors and 0.68 for surgeons), and Brier score (0.037 for junior doctors and 0.029 for surgeons).

Table 2 presents a sensitivity analysis of clinical parameters at the time of assessment by junior doctors versus colorectal surgeons. No significant differences were observed for any of the parameters, indicating no systematic differences in patient illness at the time of assessment between the assessor groups.

**Table 2:** Sensitivity analysis: comparison of clinical parameters at the time of assessment by junior doctors versus colorectal surgeons

	Junior doctors	Colorectal surgeons	*p*-value
Number of assessments (%)	6110 (90.04)	676 (9.96)	
Number of leakages assessed (%)	99 (90.19)*	12 (10.81)*	0.784
Median [IQR] postoperative day of assessment	5 [2, 6]	5 [2, 6]	0.100
Clinical parameters at time of assessment			
Heart rate (mean ± SD)	83 (15)	84 (16)	0.69
Respiratory rate (median [IQR])	14 [12, 16]	14 [12, 16]	0.941
Temperature (mean ± SD)	37.2 [0.64]	37.1 [0.70]	0.501
Systolic blood pressure (mean ± SD)	134 (21)	134 (22)	0.198

*IQR* interquartile range, *SD* standard deviation

^*^Out of total 127 anastomotic leakages

## Discussion

This large, prospective study shows that the accuracy of clinical assessment for diagnosing clinically relevant anastomotic leakage after colorectal surgery improves with clinician experience. Colorectal surgeons more accurately identified patients at risk compared to junior doctors, both in terms of discrimination and calibration. Specifically, the AUC was 0.87 (95% CI 0.82–0.92) for junior doctors and 0.95 (95% CI 0.91–0.99) (*p* = 0.02) for surgeons. Furthermore, surgeons demonstrated better calibration compared to junior doctors, as their estimated probabilities aligned more closely to the observed incidence of leakage, especially in the higher predicted probabilities. However, both groups generally tended to overestimate the actual probability. Importantly, a sensitivity analysis comparing clinical parameters at the time of assessment showed no significant differences between clinician groups, suggesting that the observed differences in diagnostic performance are unlikely to be explained by case mix. Because the intervention-based reference standard primarily captures clinically relevant anastomotic leakage (ISREC grade B/C) in the first 30 days during admission, our findings are most applicable to the detection of leakage that drives postoperative imaging, intervention, and escalation of care, rather than grade A leaks or leaks leading to readmission. These findings suggest that experience significantly enhances the clinical interpretation of subtle postoperative signs, which supports the value of senior oversight in early postoperative decision-making.

The improved diagnostic performance of more experienced clinicians may reflect their greater exposure to anastomotic leakage, a complication that occurs in up to 20% of patients. In contrast, many junior doctors may have little or no firsthand experience with anastomotic leakage, making it more challenging for them to recognize the often subtle clinical signs [18, 19]. Additionally, literature has suggested that junior doctors are less capable of detecting anastomotic leakage, due to deterioration of clinical skills compared to junior doctors in previous generations [5, 20, 21]. Conversely, repeated exposure to postoperative complications and accumulated clinical experience likely improve pattern recognition and diagnostic precision. Another plausible explanation is that experienced surgeons implicitly integrate baseline patient risk factors and intraoperative events into their postoperative clinical judgment, thereby effectively weighting prior risk together with subtle changes in the postoperative trajectory. This type of integrative, experience-based reasoning is a core component of clinical expertise and may explain the observed differences in diagnostic accuracy, despite similar bedside clinical parameters at the time of assessment. Our findings differ from earlier work suggesting no significant difference between senior and junior clinicians in diagnostic accuracy at the emergency department. However, that study primarily involved common conditions such as appendicitis or diverticulitis, which are more easily recognized regardless of clinical experience [3].

Key strengths of this study include its prospective, multicenter design and the large number of clinical assessments (> 8000 observations), which enhance both its robustness and generalizability. Importantly, clinical assessments were prospectively recorded for every individual patient, ensuring real-world applicability and reducing the risk of confounding by indication. Moreover, to our knowledge, this is the first study to evaluate the influence of clinician seniority on the diagnostic performance of clinical judgment in detecting anastomotic leakage following colorectal surgery. This provides novel insights into the value of experience in high-stakes postoperative decision-making.

Several limitations must be acknowledged. First, clinicians were not given specific guidelines on how to assign the clinical score, potentially introducing variability in scoring. Nevertheless, this reflects actual clinical practice and enhances external validity. Second, although the study included a large number of clinical assessments, individual patients were often assessed by only one clinician group (e.g., by surgeons or by junior doctors), depending on the day of the week. As a result, direct comparisons of diagnostic accuracy between clinician groups were not performed within the same patient at the same time point. To address this, a sensitivity analysis comparing clinical parameters at the time of assessment between the two groups was performed, which showed no significant differences, indicating comparable patient illness at the time of assessment between clinician groups. Third, 271 observations were excluded due to missing assessor group data, and over 1600 scores were imputed, however accounted for through multiple imputation. Fourth, in daily practice, surgeons are more often consulted when a patient deteriorates and in this case may have assessed a different case mix than the junior doctors. This could lead to bias if they more frequently reviewed patients with clearer signs of anastomotic leakage. Fifth, multiple assessments were performed in almost all patients, which introduces correlation between measurements within the same individual. This clustering reduces the statistical independence of observations and may lead to slightly overstated precision of the AUC estimates. While this reflects real-world clinical practice, where patients are reviewed multiple times, it should be considered when interpreting the results. Finally, the marked imbalance in the number of assessments between junior doctors and colorectal surgeons represents a limitation. However, this imbalance reflects routine clinical practice, in which junior doctors perform the majority of first daily postoperative evaluations, particularly during weekdays, whereas colorectal surgeons more frequently perform these assessments during weekends.

These results have important implications for clinical practice, particularly in the context of enhanced recovery after surgery pathways and early discharge programs. Given the demonstrated impact of clinical experience on diagnostic accuracy, enhanced supervision of less experienced doctors may lead to earlier diagnosis of anastomotic leakage, which in turn reduces morbidity and mortality [11]. Additionally, acknowledging that both clinician groups, especially the junior doctors, tend to overestimate the risk of anastomotic leakage, may help reduce unnecessary CT imaging. This aligns with results of a previous study, which has shown that inexperienced doctors are more likely to request unnecessary imaging [22]. While our focus was on anastomotic leakage, similar benefits of clinical experience may be expected in recognizing other postoperative complications, such as intra-abdominal abscesses or ileus. Integrating the clinical assessment of experienced staff into structured postoperative care, either through formal algorithms or decision-support tools, could improve diagnostic precision. This in turn enhances both patient safety and resource efficiency.

In conclusion, clinical experience improves the diagnostic accuracy of postoperative assessments for anastomotic leakage after colorectal surgery. While this effect likely extends to other postoperative complications, our findings clearly demonstrate the importance of experience in this specific context. These results support structured involvement of experienced clinicians and low-threshold consultation for junior doctors. Future care pathways should explicitly integrate senior input and provide targeted training to strengthen diagnostic reasoning for junior doctors in early postoperative care.

## References

[1] Mengou IV, Yakar D, Kasalak Ö, Kwee TC (2021) Towards a benchmark of abdominal CT use during duty shifts: 15-year sample from the Netherlands. Abdom Radiol 46(4):1761–1767. 10.1007/s00261-020-02818-7. (**PMID: 33078244; PMCID: PMC8096762**) 10.1007/s00261-020-02818-7 PMC8096762 33078244

[2] Elder AT, McManus IC, Patrick A, Nair K, Vaughan L, Dacre J (2017) The value of the physical examination in clinical practice: an international survey. Clin Med 17(6):490–498. 10.7861/clinmedicine.17-6-490. (**PMID: 29196348; PMCID: PMC6297700**) 10.7861/clinmedicine.17-6-490 PMC6297700 29196348

[3] Acute Abdominal Pain (AAP) Study Group (2016) Diagnostic accuracy of surgeons and trainees in assessment of patients with acute abdominal pain. Br J Surg 103(10):1343–1349. 10.1002/bjs.10232. (**PMID: 27465409.**) 27465409 10.1002/bjs.10232

[4] Young GJ, Flaherty S, Zepeda ED, Mortele KJ, Griffith JL (2020) Effects of physician experience, specialty training, and self-referral on inappropriate diagnostic imaging. J Gen Intern Med 35(6):1661–1667. 10.1007/s11606-019-05621-3. (**Erratum in: J Gen Intern Med. 2020 May 6. doi: 10.1007/s11606-020-05761-x. PMID: 31974904; PMCID: PMC7280459.**) 31974904 10.1007/s11606-019-05621-3 PMC7280459

[5] Feddock CA (2007) The lost art of clinical skills. Am J Med 120(4):374–378. 10.1016/j.amjmed.2007.01.023. (**PMID: 17398236**) 17398236 10.1016/j.amjmed.2007.01.023

[6] Turrentine FE, Denlinger CE, Simpson VB, Garwood RA, Guerlain S, Agrawal A et al (2015) Morbidity, mortality, cost, and survival estimates of gastrointestinal anastomotic leaks. J Am Coll Surg 220(2):195–206. 10.1016/j.jamcollsurg.2014.11.002. (**PMID: 25592468**) 25592468 10.1016/j.jamcollsurg.2014.11.002

[7] Koedam TWA, Bootsma BT, Deijen CL, van de Brug T, Kazemier G, Cuesta MA, COLOR COLOR II study group et al (2022) Oncological outcomes after anastomotic leakage after surgery for colon or rectal cancer: increased risk of local recurrence. Ann Surg 275(2):e420–e427. 10.1097/SLA.0000000000003889. (**PMID: 32224742.**) 32224742 10.1097/SLA.0000000000003889

[8] Krarup PM, Nordholm-Carstensen A, Jorgensen LN, Harling H (2014) Anastomotic leak increases distant recurrence and long-term mortality after curative resection for colonic cancer: a nationwide cohort study. Ann Surg 259(5):930–938. 10.1097/SLA.0b013e3182a6f2fc. (**PMID: 24045445**) 24045445 10.1097/SLA.0b013e3182a6f2fc

[9] Tonini V, Zanni M (2023) Impact of anastomotic leakage on long-term prognosis after colorectal cancer surgery. World J Gastrointest Surg 15(5):745–756. 10.4240/wjgs.v15.i5.745. (**PMID: 37342854; PMCID: PMC10277951**) 37342854 10.4240/wjgs.v15.i5.745 PMC10277951

[10] Boström P, Haapamäki MM, Rutegård J, Matthiessen P, Rutegård M (2018) Population-based cohort study of the impact on postoperative mortality of anastomotic leakage after anterior resection for rectal cancer. BJS Open 3(1):106–111. 10.1002/bjs5.50106. (**PMID: 30734021; PMCID: PMC6354192**) 30734021 10.1002/bjs5.50106 PMC6354192

[11] Kornmann VN, van Ramshorst B, Smits AB, Bollen TL, Boerma D (2014) Beware of false-negative CT scan for anastomotic leakage after colonic surgery. Int J Colorectal Dis 29(4):445–451. 10.1007/s00384-013-1815-5. (**PMID: 24356897**) 24356897 10.1007/s00384-013-1815-5

[12] den Dulk M, Witvliet MJ, Kortram K, Neijenhuis PA, de Hingh IH, Engel AF et al (2013) The DULK (Dutch leakage) and modified DULK score compared: actively seek the leak. Colorectal Dis 15(9):e528–e533. 10.1111/codi.12379. (**PMID: 24199233**) 24199233 10.1111/codi.12379

[13] Kornmann V, van Ramshorst B, van Dieren S, van Geloven N, Boermeester M, Boerma D (2016) Early complication detection after colorectal surgery (Condor): study protocol for a prospective clinical diagnostic study. Int J Colorectal Dis 31(2):459–464. 10.1007/s00384-015-2468-3 26670674 10.1007/s00384-015-2468-3

[14] von Elm E, Altman DG, Egger M, Pocock SJ, Gøtzsche PC, Vandenbroucke JP, STROBE Initiative (2007) The strengthening the reporting of observational studies in epidemiology (STROBE) statement: guidelines for reporting observational studies. Bull World Health Organ 85(11):867–872. 10.2471/BLT.07.045120. (**PMID: 12815075.**) 18038077 10.2471/BLT.07.045120 PMC2636253

[15] Rahbari NN, Weitz J, Hohenberger W et al (2010) Definition and grading of anastomotic leakage following anterior resection of the rectum: a proposal by the International Study Group of Rectal Cancer. Surgery 147(3):339–351. 10.1016/j.surg.2009.10.012 20004450 10.1016/j.surg.2009.10.012

[16] Li P, Stuart EA, Allison DB (2015) Multiple imputation: a primer. JAMA 314(18):1966–1967. 10.1001/jama.2015.15281 26547468 10.1001/jama.2015.15281 PMC4638176

[17] Tan FES, Jolani S, Verbeek H (2018) Guidelines for multiple imputations in repeated measurements with time-dependent covariates: a case study. J Clin Epidemiol 102:107–114. 10.1016/j.jclinepi.2018.06.006 29964148 10.1016/j.jclinepi.2018.06.006

[18] Kang CY, Halabi WJ, Chaudhry OO, Nguyen V, Pigazzi A, Carmichael JC et al (2013) Risk factors for anastomotic leakage after anterior resection for rectal cancer. JAMA Surg 148(1):65–71. 10.1001/2013.jamasurg.2. (**PMID: 22986932.**) 22986932 10.1001/2013.jamasurg.2

[19] McDermott FD, Heeney A, Kelly ME, Steele RJ, Carlson GL, Winter DC (2015) Systematic review of preoperative, intraoperative and postoperative risk factors for colorectal anastomotic leaks. Br J Surg 102(5):462–479. 10.1002/bjs.9697 25703524 10.1002/bjs.9697

[20] Willett LL, Estrada CA, Castiglioni A (2007) Does residency training improve performance of physical examination skills? Am J Med Sci 333:74–77 17301584 10.1097/00000441-200702000-00002

[21] Jauhar S (2006) The demise of the physical examination. N Engl J Med 354:548–551 16467540 10.1056/NEJMp068013

[22] Fouche PE, Jenkins LS, Vermeulen A (2020) Appropriateness of computed tomography and magnetic resonance imaging scans in a rural regional hospital in South Africa: a 6-year follow-up study. S Afr Med J 111(1):46–51. 10.7196/SAMJ.2020.v111i1.14860. (**PMID: 33404005**) 33404005 10.7196/SAMJ.2020.v111i1.14860

